# Facultative pupal mating in *Heliconius erato*: Implications for mate choice, female preference, and speciation

**DOI:** 10.1002/ece3.3624

**Published:** 2018-01-13

**Authors:** Timothy J. Thurman, Emily Brodie, Elizabeth Evans, William Owen McMillan

**Affiliations:** ^1^ Smithsonian Tropical Research Institute Panama City Republic of Panama; ^2^ Department of Biology and Redpath Museum McGill University Montreal QC Canada; ^3^ Department of Biology University of Puerto Rico, Rio Pedras San Juan Puerto Rico; ^4^ Department of Environmental Science and Policy University of California‐Davis, One Shields Ave Davis CA 95616

**Keywords:** *Heliconius*, mate choice, mating system, pupal mating, sexual conflict

## Abstract

Mating systems have broad impacts on how sexual selection and mate choice operate within a species, but studies of mating behavior in the laboratory may not reflect how these processes occur in the wild. Here, we examined the mating behavior of the neotropical butterfly *Heliconius erato* in the field by releasing larvae and virgin females and observing how they mated. *H. erato* is considered a pupal‐mating species (i.e., males mate with females as they emerge from the pupal case). However, we observed only two teneral mating events, and experimentally released virgins were almost all mated upon recapture. Our study confirms the presence of some pupal‐mating behavior in *H. erato*, but suggests that adult mating is likely the prevalent mating strategy in this species. These findings have important implications for the role of color pattern and female mate choice in the generation of reproductive isolation in this diverse genus.

## 1 INTRODUCTION

Animal mating systems establish which sex holds more power in mate choice (Shuster, 2009). This balance influences which traits are under sexual selection and arbitrates conflicts between the sexes. By observing mating behavior, we can begin to understand the evolutionary and ecological processes that generated the current mating system and predict how mating systems could influence evolution.

**Figure 1:**
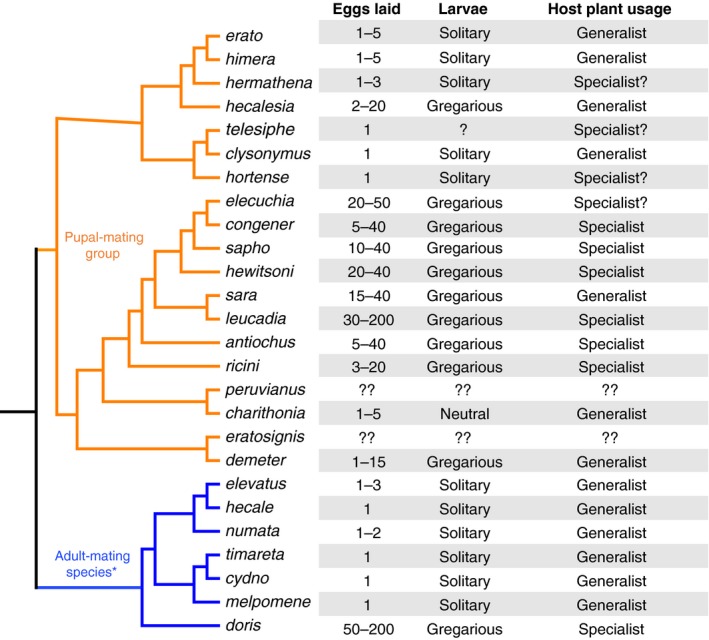
Simplified phylogeny of *Heliconius* butterflies. All members of the pupal‐mating clade are shown (in orange), but for simplicity, only a subset of adult‐mating species are presented here. Phylogenetic relationships following Kozak et al. (2015). Branch lengths are not scaled. Ecological data summarized from Brown (1981), though we note that classification of *Heliconius* as host‐plant specialists and generalists at the species level is an oversimplification (see Section 4)

The colorful, mimetic butterflies in the genus *Heliconius* are an excellent system in which to explore mating strategies and the role they play in sexual selection, sexual conflict, and speciation. In terms of mating system, *Heliconius* butterflies are traditionally classified into two groups (Figure 1). About half of *Heliconius* species are considered adult mating, the prevalent mode of mating in butterflies: males approach and court adult females, who either reject or copulate with the male (Rutowski, 1984; Scott, 1972; Walters, Stafford, Hardcastle, & Jiggins, 2012).

The other half of *Heliconius* species exhibit a mating system known as pupal mating. First described in the late 1800s, pupal mating occurs when male butterflies copulate with females as they emerge from the pupal case (Deinert, Longino, & Gilbert, 1994; Edwards, 1881; Gilbert, 1975, 1976, 1991). This is an example of sexual coercion (*sensu* Clutton‐Brock & Parker, 1995), as females are unable to reject male courtship attempts. Males may spend days waiting on the pupae and may compete with other males for space on and access to the female (Deinert et al., 1994; Edwards, 1881). In some cases, males even break the pupal case and insert their abdomen to begin mating before the female has fully emerged (Deinert et al., 1994; Gilbert, 1975; Sourakov, 2008). In this article, we distinguish between matings in which the male inserts his abdomen into the pupal case (“pharate matings”) and those in which males do not insert their abdomen, but still mate with the female during or immediately after emergence when she is unable to resist (“teneral matings”), as some may consider only pharate matings “true” pupal mating (Sourakov, 2008; Walters et al., 2012). Pupal‐mating species are monophyletic within the *Heliconius* phylogeny (Beltrán, Jiggins, Brower, Bermingham, & Mallet, 2007). Only one other species of butterfly, the Lycaenid *Jalmenus evagoras*, is known to engage in pupal mating (Elgar & Pierce, 1988), though similar pupal guarding and mating behaviors are seen in other insect orders (Thornhill & Alcock, 1983).

As a coercive mating strategy, pupal mating appears to impose serious costs on females, potentially leading to strong sexual conflict. Fiercely competing males have been observed to injure females and even knock them from the pupal case to the ground (Edwards, 1881; Gilbert, 2003). Beyond the increased risk of injury or death, pupal mating seems to eliminate females' ability to actively select the “best” mate. Lack of choice could be costly if female reproductive output is based on male quality and there is variation in male quality. This is likely in *Heliconius*, as males (of both adult‐ and pupal‐mating species) transfer a nutrient‐rich spermatophore to the female during mating that females use for egg production (Boggs & Gilbert, 1983). However, they also transfer an anti‐aphrodisiac pheromone to females to discourage remating (Estrada, Schulz, Yildizhan, & Gilbert, 2011; Gilbert, 1976; Schulz, Estrada, Yildizhan, Boppré, & Gilbert, 2008). Males can mate multiply, while female remating rates are estimated to be about 25% in adult‐mating species and pupal‐mating females are generally but not exclusively monandrous (Pliske, 1973; Walters et al., 2012).

Pupal mating may also be costly for males. Searching for and guarding pupae preclude foraging for food, and males have been observed to exhaust and starve themselves waiting on pupae (Deinert et al., 1994). Pupal mating could also influence what cues are most important for attraction and mate choice. Color pattern has long been considered an important part of species recognition and attraction in *Heliconius* butterflies (Crane, 1955). However, if males locate females as pupae when color pattern is absent or obscured, chemical or pheromonal cues from the host plant and pupae may be more important to mate choice (Estrada & Gilbert, 2010; Estrada, Yildizhan, Schulz, & Gilbert, 2010). Pupal mating is also likely to intensify male–male competition, with corresponding sexual selection on traits (e.g., wing size, olfaction, spatial memory) which would increase mating success.

Though many *Heliconius* are considered pupal‐mating species (e.g., in Brown, 1981; Beltrán et al., 2007), formal study of this behavior is mostly limited to two species. Studies of wild populations of *H. hewitsoni* and *H. charithonia* have described a suite of searching and mate‐guarding behaviors associated with pupal mating (Deinert, 2003; Deinert et al., 1994; Mendoza‐Cuenca & Macías‐Ordóñez, 2010). Insectary studies have also shown that *H. charithonia* males use host plants to find immatures and distinguish male and female pupae using chemical cues (Estrada & Gilbert, 2010; Estrada et al., 2010). However, Mendoza‐Cuenca and Macías‐Ordóñez (2010) also found evidence of adult mating in a population of *H. charithonia* with highly asynchronous female pupal emergence. Males with smaller wings, likely to be unsuccessful competing for female pupae, instead patrolled territories and were observed to mate with experimentally released adult virgin females (Mendoza‐Cuenca & Macías‐Ordóñez, 2010). This shows that, even in pupal‐mating species, other modes of reproduction may occur.

Here, we study mating behavior in a Panamanian population of the red postman butterfly, *Heliconius erato* (L. 1758, Lepidoptera: Nymphalidae). Though nominally a pupal‐mating species, *H. erato* differs from other pupal‐mating species across multiple behavioral, life history, and biogeographic axes (Brown, 1981; Beltrán et al., 2007; Walters et al., 2012; Figure 1). The published literature contains conflicting evidence about the extent to which pupal mating occurs in *H. erato*. Mating behavior has been best studied in insectaries, where some researchers have reported pupal mating (Gilbert, 1976; Muñoz, Salazar, Castaño, Jiggins, & Linares, 2010), while others observed no pupal mating (McMillan, Jiggins, & Mallet, 1997; Walters et al., 2012). Most insectary studies have treated *H. erato* as though it were an adult‐mating species by observing males' mating behavior toward either live adult females (e.g., Crane, 1955; Klein & Araújo, 2010; McMillan et al., 1997; Merrill, Chia, & Nadeau, 2014) or simulated adult females made from pinned wings or paper models (e.g., Estrada & Jiggins, 2008; Finkbeiner, Briscoe, & Reed, 2014; Merrill et al., 2014). One exception is the work of Muñoz et al. (2010), who studied both adult‐ and pupal‐mating behaviors of two color pattern races of *H. erato* which hybridize in Colombia. In their experiments, one female pupa or adult virgin female was placed in a cage with either conspecific or heterospecifics males, and matings were observed. All pupae were pupal‐mated, while only about 65% of virgin adult females mated. They found strong assortative mating by color pattern in adult matings but no evidence for assortative mating during pupal matings (Muñoz et al., 2010). Collectively, these studies have demonstrated that captive *H. erato* can perform complex courtship behaviors, that males use color pattern as a cue when approaching and courting adult females, and that mating systems could influence the degree to which populations are reproductively isolated.

Whether these adult‐mating behaviors are relevant in natural populations is unclear. To our knowledge, only one study has approached the issue. Though not a study of mating behavior, Mallet (1986) collected wild *H. erato* eggs, reared them to pupation, and placed pupae back on their host plants as part of a study to determine dispersal distance. He observed no pharate mating, though this could have been because most pupae were placed out on the day of eclosion. Studies with *H. charithonia* suggest that chemical cues emitted by host plants in response to herbivory may be important in attracting adult butterflies, and pupae alone may go undiscovered by males (Estrada & Gilbert, 2010; Mendoza‐Cuenca & Macías‐Ordóñez, 2010). However, seven of the 56 teneral females Mallet released were observed mating with wild males within a few hours of eclosion, mostly after they had moved a short distance from the pupal exuvium (J. Mallet, pers. comm.).

Given the ecological and evolutionary implications of the two mating systems in *H. erato*, it is crucial to gain a better understanding of the rate of pupal mating in the wild. Here, we investigated pupal mating in *H. erato* with two experiments. In the first, we placed larvae on experimental host plants in the field, tracked them through pupation and adult emergence, and observed their mating behavior. In the second, we released virgin females and determined their mating status upon recapture. Together, these experiments provide insight into the occurrence and relative importance of pupal mating in the wild.

## 2 METHODS

### 2.1 Experiment 1: Pupal‐mating observations

All experiments were carried out between February and May 2014 in Gamboa, Panamá, and nearby Soberanía National Park. To improve chances that pupae were discovered by males, we placed larvae, instead of pupae, on experimental plants at five sites and tracked them through pupation (see Supporting information for GPS coordinates). Second, we chose experimental sites that were within one meter of the larval host‐plant *Passiflora biflora* and near an adult food source, usually *Lantana camara*, in the hopes that our experimental sites would be quickly incorporated into the traplines, or daily routes, of adult individuals (Gilbert, 1991). Sites were between 250 m and 2.5 km apart.

At each site we hung potted *P*. *biflora* from a metal frame and applied grease and Tanglefoot (Tree Tanglefoot Co., Grand Rapids, MI) to the legs of the frame. This was an attempt to prevent ants, an important cause of larval mortality in *Heliconius*, from accessing the experimental plants (Smiley, 1985, 1986). After placing the plants, we waited 1 week before placing out larvae to allow discovery of the experimental plants.

Before releasing larvae, we attempted to catch, mark, and release all adult males at each site. Studies with *H. charithonia* have suggested that male size is correlated with mating strategy, and a bimodal distribution of male wing sizes has been interpreted as evidence of coexisting mating strategies (Mendoza‐Cuenca & Macías‐Ordóñez, 2010). Therefore, we measured each male's wing length (from the base to the tip of the left forewing). For most males, this was calculated as the average of three measurements to the nearest 0.01 mm, though some males were measured more or fewer times. In our study, wing measures were highly repeatable (see Supporting information for details).

During the experimental period, we continually released insectary‐reared third or fourth instar larvae to maintain one to six larvae per site. Our insectary stocks were derived from wild‐caught *H. erato* males and females from around the Gamboa area. Plants were replaced as needed to ensure fresh shootings for larval consumption, and larvae and plants were checked every other day until pupation. As in other Lepidoptera, late fifth‐instar *H. erato* larvae often enter a “wandering” phase as they search for a pupation site, usually on the host plant or in dry vegetation nearby (Truman and Riddiford, 1974; T. Thurman, E. Brodie, and E. Evans pers. obs.). To ensure we could locate pupae, we placed a mesh cage around the host plant of final‐day larvae and removed the cage upon pupation.

Pupae were observed daily for a period of 30 min at some point between 8:00 and 12:00 hr. During the observation period, we recorded all adult *H. erato* visits to the site and documented feeding, searching for host plants, and larval and pupal visitation behaviors of adult males, as well as their proximity to the experimental plants. On the day of emergence, pupae were observed continuously from 7:00 a.m. until the newly emerged butterfly flew from the site. Females who were not mated during this time were marked immediately after flying from the pupa to be included as experimentally released virgins for experiment 2. All statistical analyses were performed in R version 3.2.2 (R Core Team, 2013). Mixed‐effect models were fit using the R package lme4 (Bates, Mächler, Bolker, & Walker, 2015).

### 2.2 Experiment 2: Female release and recapture

We released insectary‐reared virgin *H. erato* females at three sites around Gamboa. Females were individually numbered and released on the morning of their emergence. We searched for marked females for an average of 10 hr/week, usually between 8 a.m. and 12 p.m. *H. erato* females have a strong male‐transferred odor when they are newly mated, and the transferred spermatophore can easily be felt by palpating the abdomen (Gilbert, 1976; Walters et al., 2012). Recaptured females were deemed mated if they smelled strongly of the male‐transferred odor and/or contained a palpable spermatophore. Mated females were removed from the population, while unmated females were released to be potentially recaptured.

## 3 RESULTS

### 3.1 Experiment 1: Observations of mating behavior

**Figure 2:**
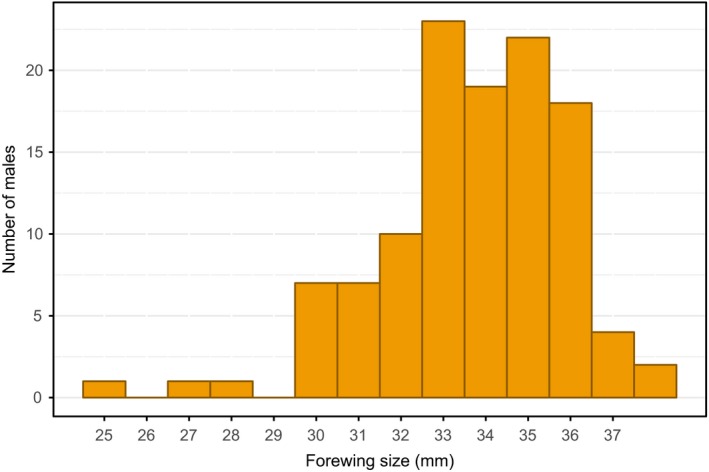
Distribution of forewing length, in mm, of *H. erato* males captured in Gamboa, Panama, during the study period. Wing sizes are binned in 1 mm intervals and corrected for differences between measurers (see Supporting information for details)

Over the course of almost one hundred field days, we caught and marked 231 wild individuals (128 males and 103 females) in the study area. We recaptured 53 males (females were not individually marked), and the vast majority (50/53) of our recaptures were at the same study area, suggesting that movement of males between our experimental sites was rare. Male forewing length was not bimodally distributed (Figure 2), which could be interpreted as evidence against the existence of multiple mating strategies (Mendoza‐Cuenca & Macías‐Ordóñez, 2010). However, wing size is plastic in *H. erato* and largely determined by larval nutrition (Rodrigues & Moreira, 2004). Thus, it is unclear whether the existence of multiple mating strategies might influence the distribution of wing sizes.

We tracked a total of 237 larvae on host plants at experimental sites. Larval mortality was high: roughly 60% of larvae died within 2 days of release, and only 43 larvae survived to pupation. During 171 half‐hour field observational bouts, adult *H. erato* entered the study sites 629 times: we identified individual marked male butterflies in roughly half of those instances. There were clear differences in adult density between sites (GLMM with date as a random factor, Poisson error distribution with log link function, site term χ^2^ = 101.54, *p *<* *2.2 × 10^−16^; Fig. S2), but at both high‐ and low‐density sites, we observed behaviors consistent with pupal mating. Specifically, we observed 99 instances of males hovering around host plants, 52 visits to pupae, and three instances of males alighting on pupae. However, we never observed multiple males competing for space on the same pupae, as has been seen in other pupal‐mating species. Some males were observed more often than others, and, perhaps unsurprisingly, these more active males accounted for most of the instances of pupal‐mating behavior. All the experimental pupae are likely to have been discovered by males in the area. Of 41 pupae living more than 2 days, all were visited by adults, including 28 cases of hovering and perching by a male.

Of the 43 pupae, six emerged as males, 11 as females, and the remainder failed to emerge or disappeared. We observed similar rates of visitation to male and female pupae, but we had little power to detect different visitation rates: most visits were to pupae that failed to emerge or disappeared, for which sex could not be determined. Of the 11 females, two were mated on the day of eclosion. In both cases, the male was not present when the female emerged, but arrived and mated with the female as she hung drying from her pupal case (i.e., teneral mating). Interestingly, these two teneral matings involved the same male and occurred on back‐to‐back days at the same site. Of the nine unmated females, two were visited by males in the hour after eclosion, but males did not initiate copulation. For the seven other females, males were not observed at the site on the morning of female eclosion. During our experiments, we also observed a marked *H. erato* male court and mate with an adult female that was not part of our experiments.

### 3.2 Experiment 2: Release and recapture of virgin females

In addition to the nine females that went unmated at our experimental sites, we released 52 insectary‐reared virgin females, for a total of 61 experimentally released virgin females. We recaptured 20 of these females, 19 of which had been mated. The sole unmated female was part of experiment 1 and was visited and courted by males upon emergence, but the males did not initiate copulation. We recaptured this female multiple times, and she was unmated on her final recapture 2 weeks after emerging.

## 4 DISCUSSION

In recent years, *Heliconius* butterflies have become an important organism for genomic studies of adaptation, speciation, and the link between these processes (Kronforst & Papa, 2015; Merrill et al., 2015; Supple, Papa, Counterman, & McMillan, 2014; Van Belleghem et al., 2017). Given the central place of *H. erato* in these studies, it is important to clarify how *H. erato* mate in natural populations. Ours is the first direct study of *H. erato* pupal‐mating behavior in the wild. Although *H. erato* is part of the pupal‐mating clade, we find that pupal mating is not obligate. Males performed some behaviors associated with pupal mating (e.g., searching host plants, visiting, and perching on pupae); however, we observed no instances of pharate mating and only two instances of teneral mating in the 11 cases in which females successfully pupated and emerged. This was not because experimental pupae went undiscovered, but instead reflects a high rate of adult mating in the population. Most experimentally released females were mated upon recapture, and we observed an adult mating at one of our experimental sites.

When we combine the evidence from studies of both captive and wild *Heliconius* pupal‐maters, there is clear variation in the propensity for pupal‐mating across the clade. In some situations, pupal mating seems dominant (Deinert et al., 1994; Gilbert, 1976; Mendoza‐Cuenca & Macías‐Ordóñez, 2010), while in others, adult mating is prevalent (McMillan et al., 1997; Walters et al., 2012, this study). What factors might promote pupal mating in some species and populations, or constrain it in others, to generate this variation?

Our behavioral observations can provide some insights into the factors which might influence pupal mating. Our experimental sites varied in the quality and abundance of both adult food sources and larval host plants. Sites with higher quality resources had both higher butterfly densities and more observations of behaviors associated with pupal mating (Table 1). Indeed, the two teneral matings we witnessed, both by the same male, occurred at a high‐density site where adult food plant was abundant throughout the experimental period. High butterfly density may promote pupal mating, as individual males may be more likely to guard pupae and attempt pupal mating as a method to outcompete other males. Abundant food, similarly, might decrease the costs of guarding pupae by making it easier for pupal‐guarding males to intermittently forage.

**Table 1:** Observed instances of male behaviors at each study site. Sites are grouped by the density of adult *H. erato* (high or low density, see main text for details). Nectar and pollen availabilities were subjectively determined by observing number and quality of flowers present at experimental site throughout the experiment. Number of observation periods varied due to variation in number of pupae and eclosing adults across sites

Nectar and pollen availabilities	High‐density sites			Low‐density sites	
	1	2	6	3	5
	High	High	Medium	Low	Low
Observation periods	59	15	37	38	41
Feeding	73	43	49	0	4
Hovering/searching experimental plants	46	5	22	17	9
Visit to pupae	12	0	15	1	2
Sitting on pupa	2	0	1	0	0
Teneral mating	2	0	0	0	0
Pharate mating	0	0	0	0	0

On the other hand, the high rates of larval and pupal mortality we found may constrain pupal mating. Only ~7% of the third and fourth instar larvae we placed out survived to adulthood, even with our attempts to control ant predation. This is similar to rates of mortality seen in experiments with adult‐mating *Heliconius*, in which only ~15%–30% of larvae survived 2 days when transplanted to their natural host plants in the wild (Merrill, Naisbit, Mallet, & Jiggins, 2013; Smiley, 1985, 1986). Such high mortality rates could make it unprofitable for males to repeatedly monitor larvae and guard pupae, any one of which has a very low probability of survival.

Previous research has shown that pupal mating is more likely when adult females emerge simultaneously (Mendoza‐Cuenca & Macías‐Ordóñez, 2010). Some characteristics of the life history of *H. erato*, both in our study population and across the *H. erato* radiation more generally, are likely to lead to asynchronous female emergence and thus constrain pupal mating. First, *H. erato* tends to lay single eggs such that larvae and pupae are solitary, whereas other pupal‐mating species lay large clutches of eggs and have gregarious larvae and pupae that develop and emerge together (Beltrán et al., 2007; Brown, 1981). Second, *H. erato* is a host‐plant generalist, while most other pupal‐mating species specialize on a single, or perhaps multiple closely related, species of *Passiflora* host plant (Benson, Brown, & Gilbert, 1975; Brown, 1981; Merrill et al., 2013). The other butterfly species that engages in pupal mating, *J. evagoras*, is also a host‐plant specialist with gregarious larvae (Elgar & Pierce, 1988).

It is important to note that classifying *Heliconius* as host‐plant specialists or generalists at the species level, as we do in Figure 1, is an oversimplification. Host‐plant usage can vary across populations within a species and may be especially dependent on abundance and diversity of local *Passiflora* or the presence of competitor species. For example, *H. melpomeme* is a generalist at the species level (Brown, 1981)*,* but in Panama, it feeds almost exclusively on *P. menispermifolia* (Merrill et al., 2013). At our study sites, *H. erato* feeds on at least three species of *Passiflora* (Merrill et al., 2013). This generalist host‐plant usage might both increase the cost of searching for pupae (as there are more possible host plants on which to find females) and decrease the chances of females emerging together in the same space, making pupal mating less likely. Further field observations and experiments will be needed to fully determine how these factors, and other variables we did not consider (e.g., sex ratios, environmental conditions, butterfly community composition), influence the mating behavior of *H. erato* and other species in the pupal‐mating clade.

Our finding that adult mating is prevalent in our studied population has important implications for mate choice, sexual selection, and diversification in *H. erato*. Our results substantiate the idea that color pattern could be an important cue for species recognition and mate choice in this species. Insectary experiments in which *H. erato* is treated as an adult‐mating species have repeatedly found that males use color pattern as a cue when approaching mates (Estrada & Jiggins, 2008; Finkbeiner et al., 2014; Merrill et al., 2014; Muñoz et al., 2010), but such captive behavior may be irrelevant if most matings in the wild are pupal. We confirm that adult mating not only occurs in the wild, but that it may be the prevalent mode of mating. Adult mating may help maintain reproductive isolation between closely related subspecies of *H. erato* which differ in color pattern (Muñoz et al., 2010), and this may drive diversification and speciation. This is consistent with the high levels of color pattern variation within *H. erato,* which displays more than 25 color patterns across South and Central America (Hines et al., 2011), while most other species in the pupal‐mating clade show much less variation throughout their range. Other highly polymorphic *Heliconius* species (e.g., *H. melpomene, H. cydno, H. numata*) are all adult mating (Beltrán et al., 2007), and color pattern has been shown to be an important cue for male mate choice in some of these species (Jiggins, Naisbit, Coe, & Mallet, 2001; Merrill et al., 2012).

Until this point, mate choice studies in *H. erato* have focused almost exclusively on male preferences. If pupal mating is prevalent, this focus on males is perhaps warranted, as females are unable to actively choose mates. It has been suggested that pupal mating may be a form of passive or indirect female choice: females mate with the winner of a male–male competition and thus may be “choosing” males with the traits that confer mating success (Estrada & Gilbert, 2010). Our results, however, suggest a more active role for female choice in *H. erato*, in which adult‐mating females may be able to accept or reject male advances. Future studies of mate choice behavior in *H. erato* should consider both male and female preferences and examine which cues, including color pattern, pheromones, or courtship display, are used by each sex when selecting mates.

Our experiments are an important step toward clarifying the extent to which *H. erato* pupal‐mate in the wild, but there is still much research to be performed. Though *Heliconius* mating behavior has been studied in insectaries for over half a century, field observations and experiments remain rare. Future work should examine the relative frequency of adult and pupal mating in other populations throughout the broad range of *H. erato*. Studies of populations that have served as the source for pupal‐mating captive populations (e.g., *H. erato adanus* from Trinidad, Gilbert, 1976) would be particularly useful in helping us understand the origin and persistence of this strange and rare mating system.

## CONFLICT OF INTEREST

None declared.

## AUTHOR CONTRIBUTIONS

TJT and EB are co‐first authors. All authors conceived and designed the experiments. TJT, EB, and EE performed experiments and analyzed data. TJT and EB drafted the manuscript with input from EE and WOM. All authors edited and approved the final manuscript.
