# Supplementary material for: Facultative pupal mating in Heliconius erato: Implications for mate choice, female preference, and speciation
Source: Ecol Evol. 2018 Jan 13;8(3):1882–9. doi: 10.1002/ece3.3624 (PMC5792586; doi:10.1002/ece3.3624)
Supplement: Supplementary file 1 [file ECE3-8-1882-s001.pdf]

Supplemental material for

Facultative pupal mating in *Heliconius erato*: implications for mate choice, female preference and speciation

Supplementary image

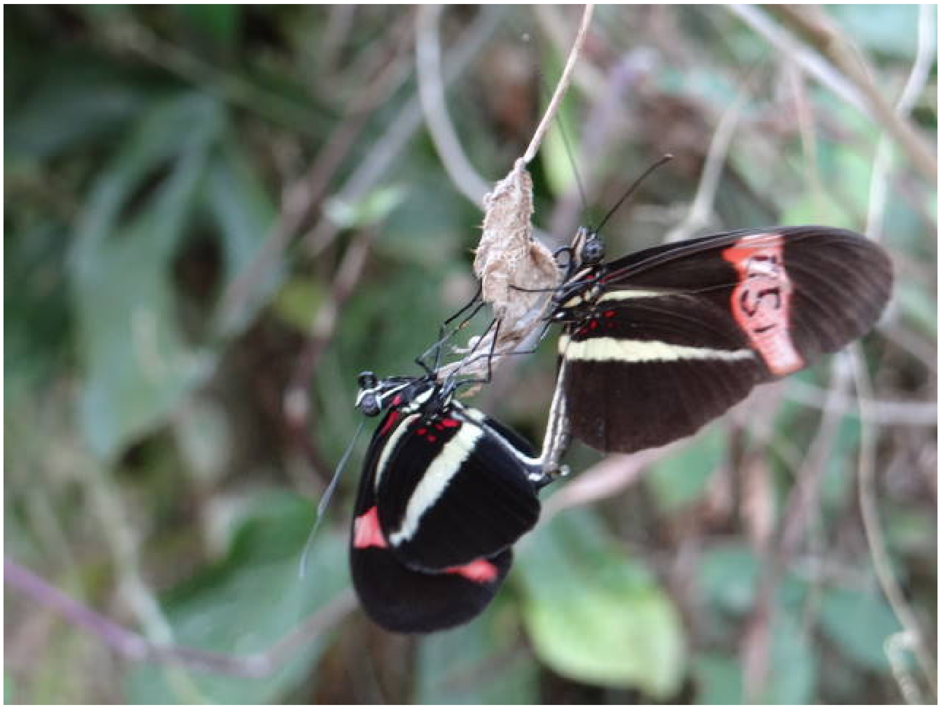

Figure S1- A teneral pupal mating occuring at site 1 between a marked male (#051) and an experimantal female (unmarked). Image courtesy of Ingo Wedemeyer, used with permission.

Location of study sites

Experiment 1		Experiment 2	
Site	GPS coordinates	Site	GPS coordinates
1	9 7.254 N, 79 42.217 W	Bldg 183	9 6.990 N, 79 41.890 W
2	9 7.273 N, 79 41.632 W	Pipeline road	9 7.281 N, 79 42.952 W
3	9 6.843 N, 79 41.889 W	Insectaries	9 7.190 N, 79 42.075 W
5*	9 7.263 N, 79 42.964 W		
6	9 7.217 N, 79 42.004 W		

*A site 4 was started but removed from the experiment after no butterflies were sighted during the first few weeks.

Table S1- GPS coordinates of study sites

Comparison of density across sites

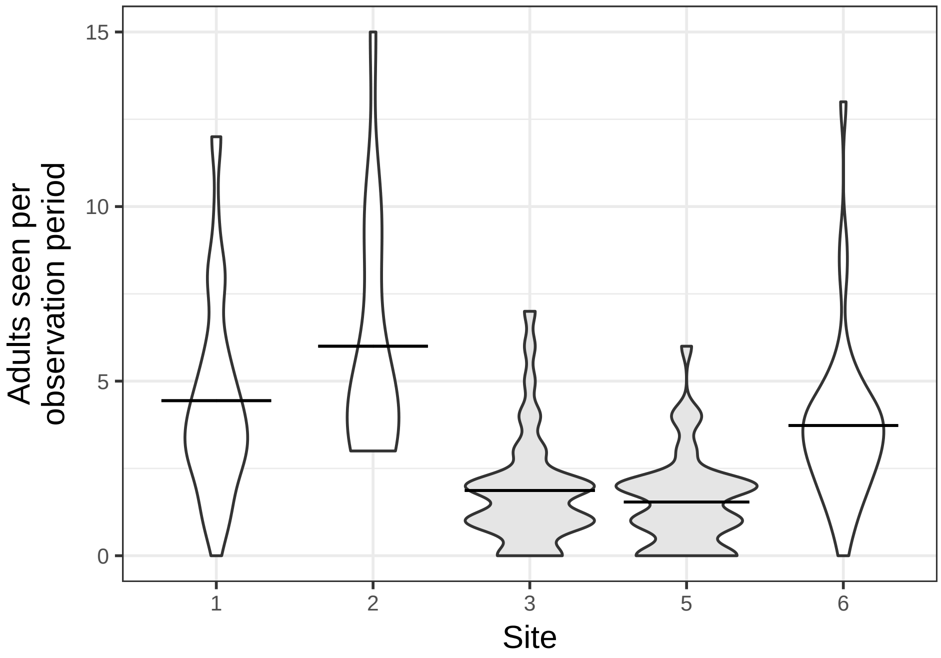

Figure S2- Comparison of adult density across study sites. Horizontal lines show the mean number of adults seen per observation period for each site, and the violin plots show the full distribution across the experimental period. Adult density differed significantly between sites (see main text). Low-density sites are filled with grey, high-density sites are unfilled.

Measurements of forewing length

Repeatability

We estimated the repeatability of our forewing length measurements following Quinn and Keough (2002) and Whitlock and Schluter (2009). Repeatability is equal to:

$$\frac{\sigma_\alpha^2}{\sigma_\alpha^2 + \sigma_E^2} \tag{1}$$

where σ_α^2 is the variance among groups (here the groups are the individual butterflies) and σ_E^2 is the variance within groups (Whitlock and Schluter 2009). Within-group variance is estimated from the mean-squared error (or residual) term of a single-factor, random-effects ANOVA in which wing length is the response variable and male ID is the factor (Table S2, Whitlock and Schluter 2009). Because individuals were measured a different number of times, we estimated σ_α^2 as:

$$\frac{\text{MS}_{\text{groups}} - \text{MS}_{\text{residual}}}{(\sum n_i - \sum n_i^2 / \sum n_i)/(p-1)} \tag{2}$$

where n_i is the number of measurements of individual i and p is the number of individuals (Quinn and Keough 2002). We measured 173 butterflies more than once, for a total of 644 measurements (mean = 3.7 measures per butterfly, mode = 3). For our data, $\sigma_\alpha^2 = 4.94$, $\sigma_E^2 = 0.12$, and repeatability was quite high (0.98).

Term	df	Sum. Sqs.	Mean Sqs.	F	P
Individual	172	2806.5	16.3	136.7	4.94×10^{-32}
Residuals	471	56.2	0.1		

Table S2- F-table of ANOVA for estimating repeatability

Measurer effects

Two authors (TT and EB) performed all measurements of male forewing length. We tested for a significant effect of measurer on male forewing length using a linear mixed model implemented in the R package `lme4` (Bates et al. 2015). For the subset of males that were measured by both TT and EB, we fit a linear mixed model with forewing length as the response variable, measurer as the predictor variable, and male ID as a random effect. We found a significant effect of measurer ($\chi^2_1 = 32.52$, $p < 0.001$): on average, TT measured butterflies as being .22mm smaller than EB.

However, this measurer effect does not influence our conclusions about the distribution of male wing sizes. Figure 2 in the main text presents the distribution of male forewing sizes with measures corrected by adding .22mm to all measurements performed by TT. However, we find a similar distribution and no strong evidence for bimodality in the uncorrected measures (Figure S3) and in measures corrected by subtracting 0.22mm from all measurements performed by EB (Figure S4).

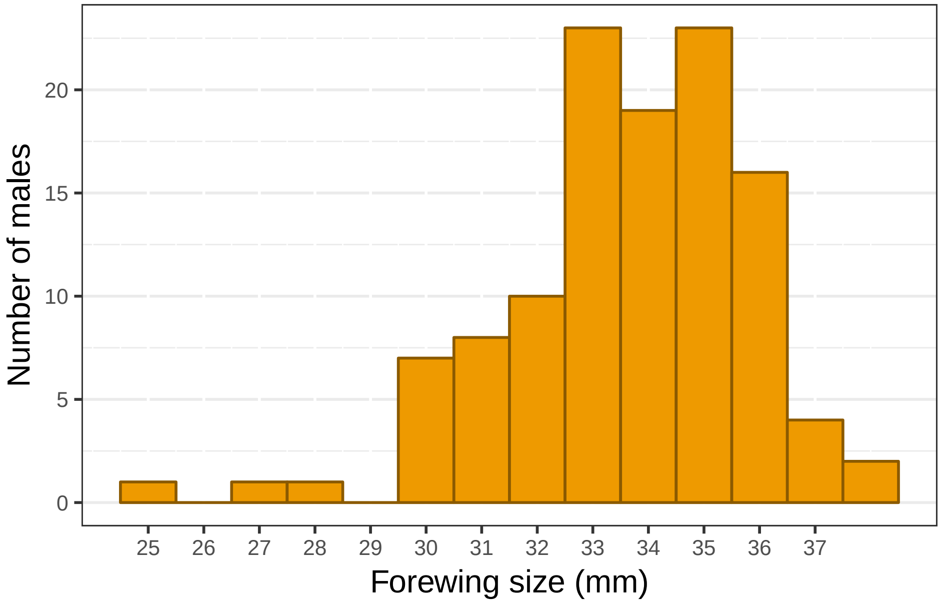

Figure S3- Uncorrected distribution of male forewing sizes.

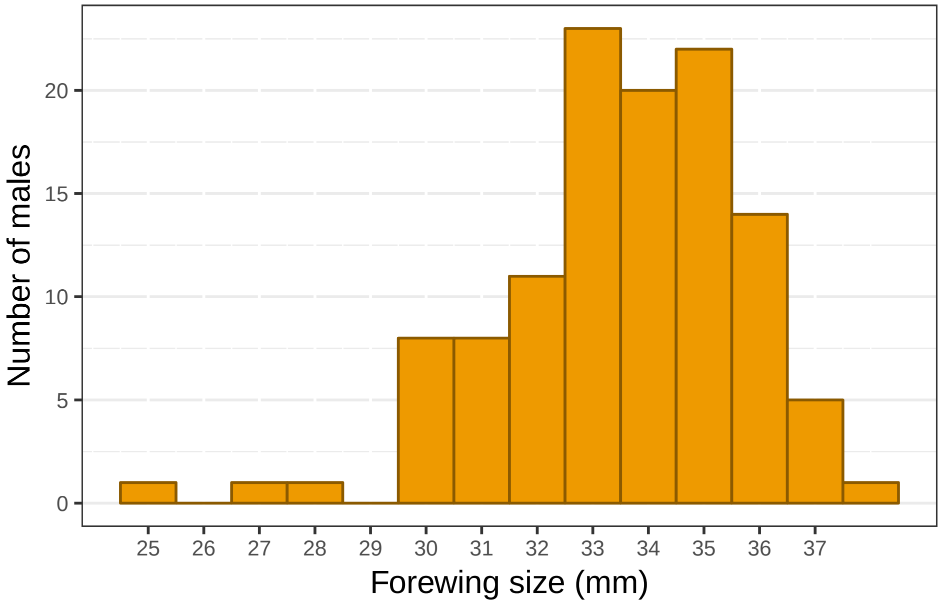

Figure S4- Distribution of male forewing sizes, corrected by subtracting 0.22mm from butterflies measured by EB.

References

Bates, D., M. Mächler, B. Bolker, and S. Walker. 2015. Fitting linear mixed-effects models using lme4. Journal of Statistical Software 67.

Quinn, G. P., and M. J. Keough. 2002. Experimental design and data analysis for biologists. Cambridge University Press, Cambridge, UK.

Whitlock, M. C., and D. Schluter. 2009. The analysis of biological data. Roberts; Co. Publishers, Greenwood Village, CO.
